# Surgically treated acromegaly patients have a similar quality of life whether controlled by surgery or requiring additional medical therapy (QuaLAT Study)

**DOI:** 10.1007/s11102-021-01153-4

**Published:** 2021-05-12

**Authors:** Muhammad Fahad Arshad, Oluwafunto Ogunleye, Richard Ross, Miguel Debono

**Affiliations:** 1 Department of Endocrinology and Diabetes, Sheffield Teaching Hospitals, Glossop Road, Sheffield, S10 2 JF UK; 2 Department of Oncology and Metabolism, University of Sheffield, Sheffield, UK

**Keywords:** Acromegaly, Quality of life, Trans-sphenoidal surgery, Pituitary tumours

## Abstract

**Purpose:**

There is no consensus on quality of life (QOL) in patients with acromegaly requiring medical treatment after surgery compared with those achieving remission by surgery alone.

**Methods:**

QuaLAT is a cross-sectional study comparing QOL in surgery-only treated acromegaly patients versus those requiring medical treatment post-surgery. Patients attending clinics were identified and divided into—Group 1: patients who had surgery only and were in biochemical remission, Group 2: all patients on medical treatment post-surgery, Group 3: patients from Group 2 with biochemical control. Participants were asked to fill three questionnaires; Acromegaly Quality of Life Questionnaire (ACROQOL), 36-Item Short Form Survey (SF36), and Fatigue Severity Scale (FSS).

**Results:**

There were 32 patients in Group 1 and 25 in Group 2. There was no difference in QOL scores between groups 1 and 2, as measured by ACROQOL (mean difference [MD] = − 2.5, 95% CI − 16.6 to 11.6; p = 0.72), SF36v2 [Physical component score (PCS) MD = − 4.9, 95% CI − 10.9 to 1.2; p = 0.12; mental component score MD = − 3.0, 95% CI − 10.5 to 4.4; p = 0.44], or FSS (MD = − 0.004, 95% CI − 1.14 to 1.33; p = 0.1). Comparison between groups 1 and 3 however showed that PCS (and 3 subdomains) was significantly better in group 3 (MD = − 8.3, 95% CI − 14.8 to -1.8; p = 0.01). All three QOL scores were lower when compared with healthy controls.

**Conclusions:**

Medical treatment not only achieves a QOL comparable to surgery, it may also be associated with better QOL in physical subdomains. When compared with healthy controls, QOL remains worse in treated acromegaly patients compared to controls.

**Supplementary Information:**

The online version contains supplementary material available at 10.1007/s11102-021-01153-4.

## Introduction

Acromegaly results from excessive secretion of growth hormone (GH) from tumours usually originating from pituitary somatotroph cells. The excess GH then stimulates the liver to produce insulin-like growth factor-1 (IGF-1), which causes most of the clinical manifestations of acromegaly [1]. Although the incidence of acromegaly has increased, it is a rare disease with an estimated prevalence of 8.6 cases per 100,000 [2]. For any chronic disease, improvement in the quality of life (QOL) is an important patient-related health outcome goal [3]. Different acromegaly treatment modalities have resulted in a clear reduction in mortality and morbidity [4] and it is well established that symptoms and QOL improve during acromegaly treatment [5], but in a relatively recent systematic review it was concluded that there is no overall consensus if any particular treatment is superior to the other in improving QOL [6].

The first-line treatment for acromegaly is trans-sphenoidal surgery (TSS), to remove or debulk the tumour [7]. However, if surgery is unable to achieve disease remission, medical treatment, such as somatostatin receptor ligands (SRL), dopamine agonists (DA), and pegvisomant are recommended to achieve biochemical control. Conventional radiotherapy or stereotactic radiosurgery (STRS) is considered if medical treatment is ineffective or intolerable and to treat residual tumour. While surgery can achieve remission in two-thirds of the cases [8], a significant proportion of patients will require some form of medical treatment afterwards to achieve hormonal control. Unfortunately irrespective of biochemical control health related quality of life remains impaired [6].

QoL reflects the subjective perception of health and effects of a disease. QOL in acromegaly has been a subject of interest in several published studies. Various validated questionnaires have been used to assess QOL in these patients. The most popular of these is Acromegaly Quality of Life Questionnaire (ACROQOL) developed by Webb et al. in 2002, which is a validated questionnaire for acromegaly patients [9]. Several other questionnaires have been used in various studies such as Patient-Assessed Acromegaly Symptom Questionnaire (PASQ) [10], and several other generic questionnaires such as 36-Item Short Form Survey (SF36) [11] and EuroQol-5 Dimensions (EQ-5D) [10]. Assessing the effects of symptoms and QOL is essential when looking at treatment outcomes [5].

When studies have assessed QOL in acromegaly the focus seems to be either on the effect of surgery [11, 12] or medications alone [13–15] or comparison of various medication combinations [16, 17]. It is still not clear if there is any difference in QOL among those who achieve remission by surgery only and those who require additional medical treatment post-surgery. Our literature review identified only one cross sectional study which looked at this question in the Japanese population [18]. They found that QOL is worse in medically treated patients, but this was a relatively small sized study (n = 26). We also found two older studies which directly compare QOL between those treated with surgery and those receiving medical treatment longitudinally both as first line treatment [19, 20]. These studies showed that the QOL was not different between the two groups, despite having improved from baseline. Another study from France showed that there was no difference in QOL among controlled patients whether treated with surgery ± medications or medications alone, however, among uncontrolled patients one subscale (psychological appearance) was better among surgical patients [21]. A significant proportion of patients will require some form of medical treatment post-surgery to control their IGF-1 levels. However, there is no clear consensus in the published literature on how the QOL in this group compares with those who achieve remission by surgery alone.

We therefore carried out a study to assess whether QOL between patients who have achieved remission post-surgery varies when compared to patients who receive medical treatment post-surgery.

## Methods

### Subjects

QuaLAT (*Qua*lity of *L*ife after *A*cromegaly *T*reatment) is a cross-sectional study carried out at the Department of Endocrinology, Sheffield Teaching Hospitals NHS Foundation Trust, a tertiary referral centre where all patients, aged 18 or above, with a histological diagnosis of a GH producing pituitary adenoma (diagnosed at least 6 months ago) were identified via our database. Exclusion criteria included having only non-surgical treatment for acromegaly, inability to provide informed consent, having a dire prognosis, or severe comorbidities unrelated to acromegaly which according to investigators may confound results. After exclusions, preliminary data for the remaining patients were collected to identify those who underwent trans-sphenoidal surgery (TSS) and went into disease remission biochemically (Group 1); that is achieved IGF1 levels within the age-and sex-specific reference range and a growth hormone suppressed level < 0.3 µg/L post OGTT and/or a growth hormone day curve average < 1.6 µg/L. Those who did not achieve biochemical remission after surgery and therefore required further medical treatment to control the disease were included in the other group (Group 2), irrespective of their biochemical status. There were some patients in Group 2 who had their last IGF-1 values and growth hormone levels outside the reference range. To avoid any potential confounding due to this factor, another group (Group 3) was crafted for the purpose of analysis, including only those patients from Group 2 who had controlled biochemistry.

The study was registered with Sheffield Teaching Hospitals research and development department (Reference STH20344). The study was approved by the Yorkshire and the Humber Research Ethics Committee (REC reference 19/YH/0373).

### Study design and aims

In this study we aimed to compare the quality of life in those who were treated with surgery only vs those treated with surgery and medications for acromegaly, at a single tertiary care centre.

Eligible patients were approached via telephone and informed about the study. The willing participants were then posted the patient information sheet, a consent form, and the QOL questionnaires with two return envelopes to post back consent and questionnaires separately to maintain confidentiality. Phone numbers to contact the research team were provided in the patient information sheet, in case they had any additional questions or concerns.

Those who did not reply back within 28 days were given another phone call to check if they are still interested in participating in the study. If the interest was expressed once again, they were given another 28 days to send the documents. If the research team was unable to contact the patients, or the documents were not received within 28 days, it was deemed that the patients were no longer interested and therefore removed from the study.

### Quality of life measurement

All the recruited patients were asked to fill three questionnaires to measure their QoL. One of these was a disease-specific questionnaire (ACROQOL) and the other two were generic questionnaires; SF36 and Fatigue severity scale (FSS). The permissions to use ACROQOL and SF36v2 were obtained prior to the study, while this was not required for FSS as it was freely available to use.

1. **ACROQOL**

ACROQOL consists of 22 questions, each having five options of responses scoring 1–5. The maximum score that can be reached is 110 (100%) which reflects the best QoL, with 22 (0%) being the lowest possible score. The score is calculated by the following equation [22]:

$$\frac{(X) - L}{{H - L}} \times 100$$

Where L is the lowest score of the subdomain of interest and H is the highest score of the same subdomain. There are two main categories for these questions: physical and psychological function, with the latter further subdivided into areas related to appearance and personal relationships.

(2) **Short form 36 (SF 36)**

The SF36 is a questionnaire widely used for assessing health-related QoL. It is not disease-specific but has been used in acromegaly patients previously [11]. It consists of eight scales: physical functioning (PF), role-physical (RP), bodily pain (BP), general health (GH), vitality (VT), social functioning (SF), role-emotional (RE), and mental health (MH). The first four scales are related to physical health with the last four related to mental health. Like ACROQOL, final score is a percentage score with higher scores reflecting better QOL and vice versa. Calculation of the score is done in two steps: first pre-coded numeric values as assigned to the scores recorded by the participant according to the scoring key and second, the items in same scale are averaged together to create the eight scale scores [23].

(3) **Fatigue severity scale (FSS)**

FSS is also a generic questionnaire, which consists of 9 questions with the patients score from 1 to 7 (1 means strong agreement and 7 means strong disagreement) assessing a specific acromegaly related symptom and its consequences. The questions evaluate the effects of fatigue on subject’s motivation, exercise, physical functioning, interference in work, family and social life. The total score is calculated by either determining the mean of all scores. Unlike the first two questionnaires, higher score signifies worse QOL.

The demographic, treatment and biochemical data were collected for the successfully recruited patients. This was done by reviewing patients’ electronic and paper case notes. In addition to age, gender, and ethnicity, the demographic data included the presence or absence of various co-morbidities often linked with acromegaly. The treatment data included the date of surgery, type of medications used to treat acromegaly (if any), use of conventional radiotherapy or stereotactic radiosurgery, and current hormone requirements. Latest IGF-1 values within the last 12 months of filling the questionnaires were recorded. All the IGF-1 values were measured using validated radioimmunoassay (Mediagnost GmBH, Reutlingen, Germany).

### Sub analysis

To assess how our final results related to the normal healthy population we compared our results from all of the three questionnaires to those published in the literature. For this comparison, we selected studies which reported QOL in a large number of healthy participants and the population demographics were largely similar to our study groups and that reported the mean and standard deviation.

Our literature search identified only one study that reported the ACROQOL scores in a healthy non-diseased population as controls to validate their results [24]. The study participants in this study were Spanish and obese [body mass index (BMI) > 30 kg/m^2^], however the mean BMI was not provided. In case of SF-36, we selected the study reported by Jenkinson et al. [25] which reported scores from a very large group from the UK population (n = 8889) to test its reliability and consistency. Lastly, for FSS, the study reported by Ongre et al. [26] was chosen. This study reported FSS scores from 170 control participants from Norway and compared with scores from patients with Parkinson’s disease.

### Statistical analysis

All of the data were analysed using SPSS version 25 and GraphPad. Rates and percentages were calculated for categorical data. Comparison between continuous variables were summarised as mean difference (MD) and 95% confidence intervals (CI) of the differences as well as means and standard deviations (SD). Differences between the groups were tested by an independent sample T-test. A p-value of < 0.05 was considered significant for the study and two-tailed tests were used. The differences in quality of life scores between three groups were analysed by ANOVA followed by Fisher’s least significance difference (LSD) test for post-hoc analysis. The differences in QOL scores between study cohort and healthy population was performed by independent sample T-test using GraphPad online calculator (https://www.graphpad.com/quickcalcs/ttest1.cfm).

## Results

**Fig. 1:**
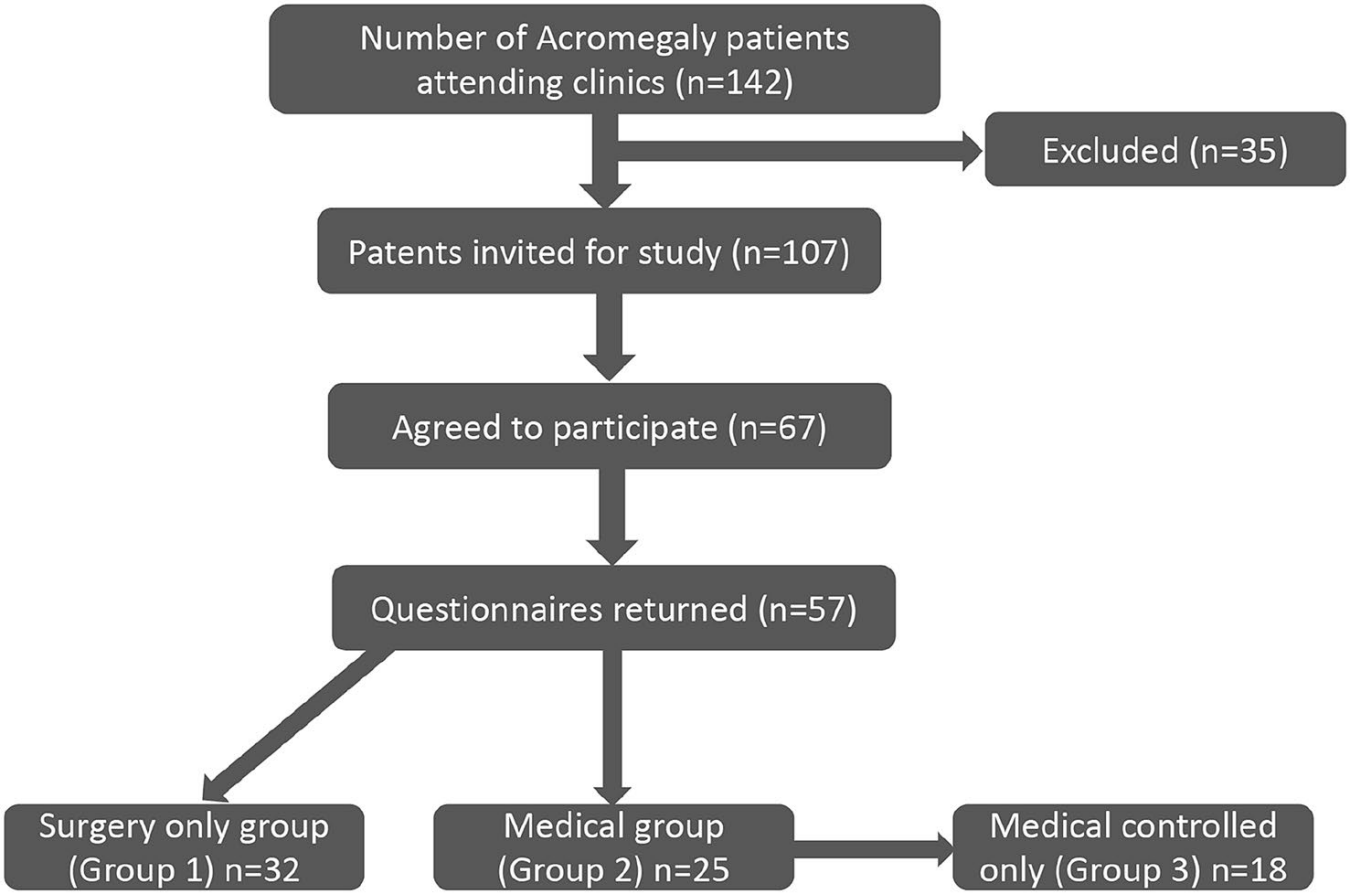
Schematic representation of study illustrating how the final number of patients were reached in groups 1 (surgery only), 2 (surgery followed by medications), and 3 (surgery followed by medications and in biochemical remission)

The total number of acromegaly patients who were attending Sheffield Teaching Hospital clinics identified via our database was 142. Those who did not meet the inclusion criteria were excluded and the remaining (n = 107) were invited for the study. Of these, 67 patients were successfully approached and showed interest to participate. These patients were then posted the study documents including questionnaires. At the end of the study we received completed questionnaires from 57 participants, who were included in the final analysis. 32 participants had only surgical treatment and were in remission post-surgery (Group 1), while the remaining 25 patients had required some form of medical therapy after surgery (Group 2). Since there were some patients in this Group 2 who were still biochemically uncontrolled based on IGF1 levels above the normal range despite treatment, a third medically controlled only group was created for the purpose of analysis (Group 3) (Fig. 1).

Table 1 summarises the comparison of baseline demographic, treatment and biochemical data between the three groups. All of the patients in this study were Caucasians. There were no significant differences between Group 1 and 2 in age, duration of disease, gender distribution, use of conventional radiotherapy, or current requirement of hormonal treatments. Most of the patients in both groups were on single hormone replacement (12/14 in group 1 and 10/12 in group 2). The hormonal treatments were adequately replaced as patients are under regular endocrine follow up. More patients in the medical group underwent STRS, as one might expect. Also, there were a few uncontrolled patients in group 2 (n = 7), as mentioned earlier.

**Table 1:** Table showing baseline demographic, treatment and biochemical data comparison between the three groups

	Group 1 (Surgical) n = 32	Group 2 (Medical) n = 25	Group 3 (Medical controlled only) n = 18	P value groups 1 and 2	P value groups 1 and 3
Mean age (years) ± SD	61.0 ± 11.3	56.7 ± 13.4	56.3 ± 12.4	0.20	0.19
Mean duration of disease (years) ± SD	9.9 ± 7.1	11.1 ± 8.5	11.9 ± 9.3	0.58	0.43
Radiotherapy (%)	3/32 (9%)	6/25 (24%)	5/18 (28%)	0.13	0.09
Stereotactic radiosurgery (STRS) (%)	3/32 (9%)	8/25 (32%)	6/18 (33%)	0.03*	0.03*
Hormone requirement (%)	14/32 (44%)	12/25 (48%)	10/18 (56%)	0.75	0.42
Gender (F/M)	19/13	10/15	6/12	0.15	0.07
Controlled v Uncontrolled	Controlled = 32 Uncontrolled = 0	Controlled = 18 Uncontrolled = 7	Controlled = 18	0.001*	-
Hypertension (%)	20/32 (63%)	16/25 (64%)	12/18 (67%)	0.91	0.77
Type 2 diabetes mellitus (%)	7/32 (22%)	7/25 (28%)	6/18 (33%)	0.59	0.38
Mental health disorder (%)	11/32 (34%)	11/25 (34%)	7/18 (39%)	0.46	0.75
Obstructive sleep apnea (%)	4/32 (13%)	3/25 (12%)	2/18 (11%)	0.95	0.89
Dyslipidaemia (%)	14/32 (44%)	10/25 (40%)	8/18 (44%)	0.78	0.96
Osteoarthritis (%)	6/32 19%)	6/25 (24%)	5/18 (28%)	0.63	0.46
Arrhythmias (%)	2/32 (6%)	2/25 (8%)	1/18 (6%)	0.80	0.92
Valvular heart problems (%)	1/32 (6%)	1/25 (4%)	0/18 (0%)	0.86	0.45
IHD/Heart Failure/Cardiomyopathy (%)	1/32 3%)	2/25 (4%)	1/18 (6%)	0.41	0.67
Bowel cancer (%)	1/32 (3%)	0/25 (0%)	0/18 (0%)	0.37	0.45

*SD* standard deviation, *F* Females, *M* Males, *IHD* Ischaemic heart disease

Groups 1 and 3 were similar with the exception of STRS. There were more males in the Group 3 (n = 12/18; p = 0.07). There was no significant difference in the incidence of other acromegaly related conditions [hypertension, T2DM, ischaemic heart disease, cardiomyopathy, heart failure, cardiac arrhythmias, obstructive sleep apnoea, mental health disorders such as anxiety or depression, dyslipidaemia (or current use of statins or fibrates), valvular heart disease (at least moderate aortic or mitral regurgitation), and bowel cancer] across all of the groups. The majority of patients in the medical group were on SRLs either alone (n = 16) or in combination with cabergoline (n = 1). Five patients were on cabergoline alone, and three patients were on pegvisomant treatment.

There was no difference in QOL scores between our main groups 1 and 2, as measured by all of three questionnaires (Table 2). Further comparison between subdomains and individual questions for all questionnaires (data not shown here) was also non-significant. However interestingly, when only medically controlled patients (Group 3) were compared with those in the surgical group, physical component score (PCS) of the SF36 questionnaire (and three of its subdomains: PF [MD = − 19.2, 95% CI − 37.2 to − 1.2; p = 0.04], RP [MD = − 22.8, 95% CI − 42.1 to − 3.5; p = 0.02], BP [MD = − 20.5, 95% CI − 36.3 to − 4.7; p = 0.01]) was significantly better in the medically controlled only patients (PCS MD = − 8.3, 95% CI − 14.8 to − 1.8; p = 0.014). This trend was also noted in the ACROQOL physical domain however the p value fell just short of significance (MD = − 15.4, 95% CI − 33.1 to 2.2; p = 0.09). A multivariate model including various variables (age, gender, duration of disease, hormone replacement, radiotherapy) also confirmed that while there was no difference between groups 1 and 2, the PCS of group 3 (and two of its subdomain [RP and BP] remained significantly better than group 1 [p = 0.04]).

**Table 2:** Comparison of QOL scores between surgical and medical group, calculated using ANOVA and Fisher’s least significance difference (LSD) test for post-hoc analysis

	Group 1 (Surgical) n = 32	Group 2 (Medical) n = 25	Group 3 (Medical controlled only) n = 18	Mean difference (95% CI) groups 1 and 2	P value groups 1 and 2	Mean difference (95% CI) groups 1 and 3	P value groups 1 and 3
Mean total ACROQOL ± SD	51.3 ± 27.1	53.8 ± 25.5	57.1 ± 27.3	− 2.5 (− 16.7 to 11.7)	0.72	− 5.8 (− 21.4 to 9.9)	0.47
ACROQOL physical domain	46.4 ± 28.3	54.8 ± 30.8	61.8 ± 31.2	− 8.4 (− 24.4 to 7.6)	0.30	− 15.4 (− 33.1 to 2.2)	0.09
ACROQOL psychological/appearance domain	49.7 ± 30.2	45.3 ± 24.7	47.2 ± 26.2	4.4 (− 10.4 to 19.1)	0.56	2.4 (− 13.8 to 18.7)	0.77
ACROQOL psychological/personal relations %	63.5 ± 28.0	62.4 ± 27.4	63.1 ± 28.0	1.1 (− 13.7 to 15.9)	0.89	0.4 (− 15.9 to 16.7)	0.96
FSS mean score ± SD	4.4 ± 2.1	4.4 ± 2.1	4.0 ± 2.2	− 0.0 (− 1.1 to 1.1)	0.10	0.4 (− 0.9 to 1.7)	0.52
SF36 physical component score (PCS)	40.6 ± 10.3	45.4 ± 12.3	48.8 ± 11.8	− 4.9 (− 11.0 to 1.2)	0.12	− 8.3 (− 15.0 to − 1.5)	0.02*
Physical functioning (PF)	56.9 ± 29.6	69.0 ± 31.2	76.1 ± 31.5	− 12.1 (− 28.4 to 4.2)	0.14	− 19.2 (− 37.2 to − 1.2)	0.04*
Role-physical (RP)	48.0 ± 29.5	62.5 ± 34.9	70.8 ± 34.8	− 14.5 (− 32.0 to 3.0)	0.10	− 22.8 (− 42.1 to − 3.5)	0.02*
Bodily pain (BP)	49.0 ± 22.6	60.9 ± 31.4	69.5 ± 27.2	− 11.9 (− 26.2 to 2.4)	0.10	− 20.5 (− 36.3 to − 4.7)	0.01*
General health (GH)	41.2 ± 25.7	48.7 ± 32.8	55.6 ± 35.1	− 7.5 (− 23.7 to 8.8)	0.36	− 14.4 (− 32.3 to 3.5)	0.11
SF36 mental component score (MCS)	39.3 ± 12.0	42.3 ± 15.8	43.7 ± 15.7	− 3.0 (− 10.7 to 4.6)	0.44	− 4.4 (− 12.9 to 4.1)	0.30
Role-emotional (RE)	57.3 ± 31.2	65.0 ± 34.9	71.3 ± 31.9	− 7.7 (− 25.1 to 9.7)	0.38	− 14.0 (− 33.2 to 5.2)	0.15
Vitality (VT)	34.3 ± 23.3	39.8 ± 31.0	44.4 ± 32.9	− 5.5 (− 20.8 to 9.8)	0.48	− 10.2 (− 27.0 to 6.7)	0.23
Mental health (MH)	52.6 ± 24.8	62.6 ± 29.3	65.0 ± 30.5	− 10.0 (− 24.9 to 4.9)	0.19	− 12.4 (− 28.9 to 4.0)	0.14
Social functioning (SF)	58.2 ± 30.7	67.0 ± 32.9	71.5 ± 32.9	− 8.8 (− 25.8 to 8.2)	0.31	− 13.3 (− 32.1 to 5.4)	0.16

### QOL in acromegaly patients’ vs healthy population

**Fig. 2:**
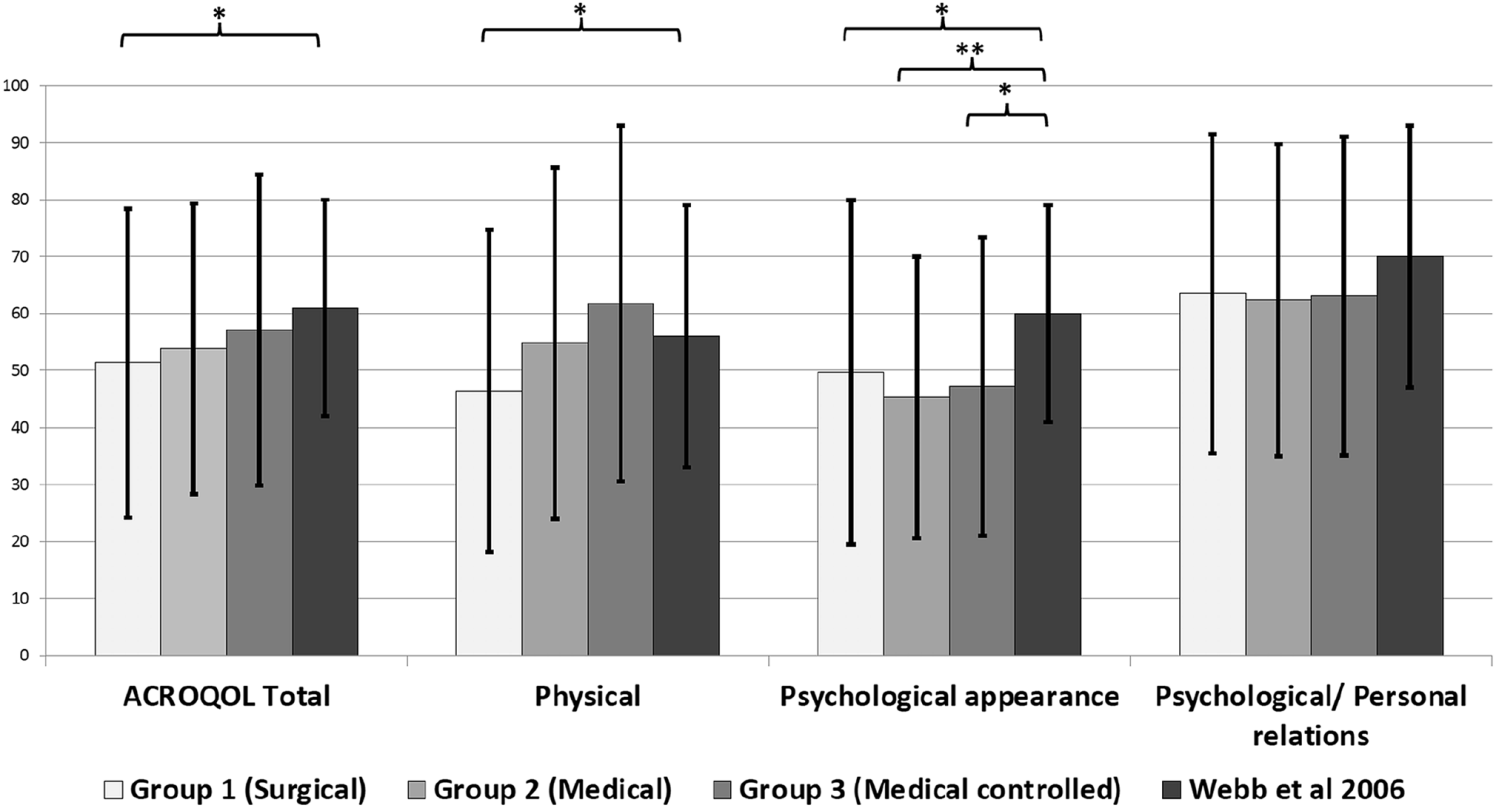
Comparison of mean ACROQL total and domain scores (with standard deviation) of acromegaly groups with healthy and obese Spanish population (asterisk symbol representing significant differences between groups; ***p < 0.001, **p < 0.01, *p < 0.05)

**Fig. 3:**
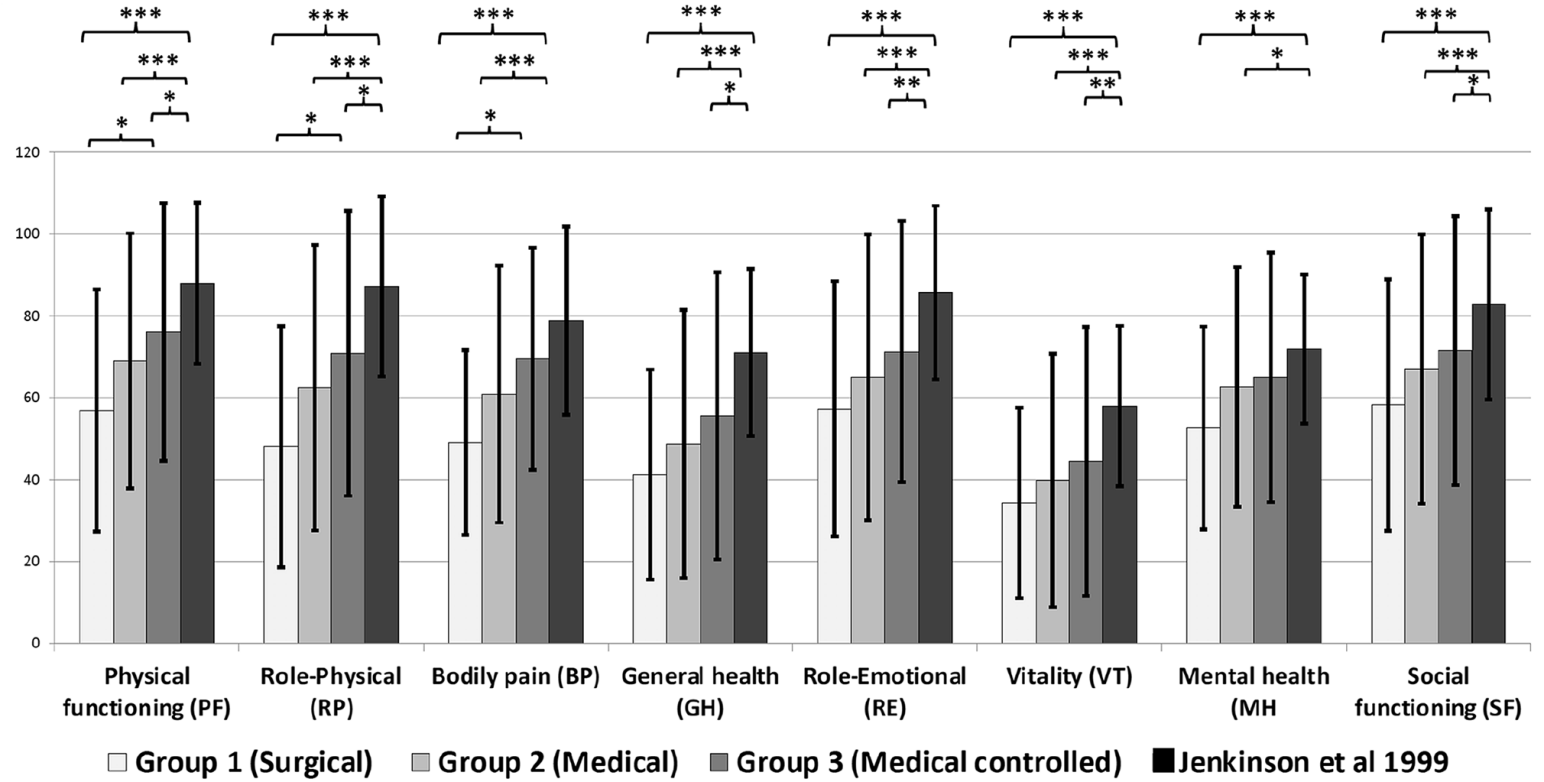
Comparison of mean SF36 subdomain scores (with standard deviation) of acromegaly groups with healthy controls (asterisk symbol representing significant differences between groups; ***p < 0.001, **p < 0.01, *p < 0.05)

**Fig. 4:**
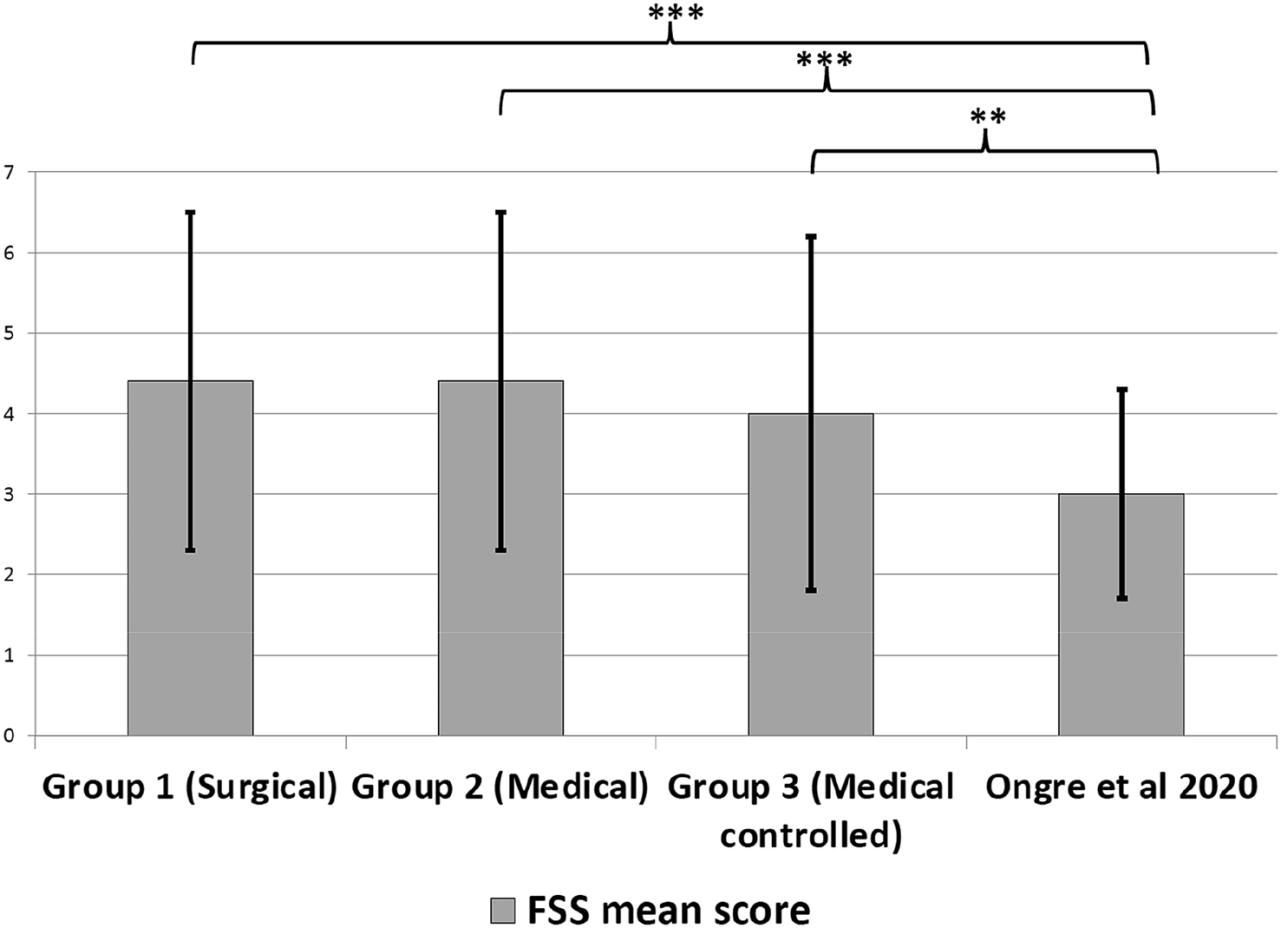
Comparison of mean FSS scores (with standard deviation) of acromegaly groups with healthy controls (asterisk symbol representing significant differences between groups; ***p < 0.001, **p < 0.01, *p < 0.05)

Figure 2 below shows the comparison of our ACROQOL scores with 157 healthy but obese controls from a Spanish population as reported by Webb et al. [24]. It can be seen that the difference in QOL is mainly in the psychological dimensions. Analogous comparisons of SF36 subdomains [25] and FSS scores [26] also mirrors the inferior QOL in all treated groups in our study (Figs. 3 and 4).

## Discussion

Our study shows that patients who had medical treatment after surgery have a similar QOL when compared to patients with acromegaly who are in remission after surgery alone. Nonetheless, while treatment improves the QOL, it remains worse in acromegaly treated patients compared to the healthy population. Our findings highlight the important role medical treatment has in patients with uncontrolled acromegaly but also demonstrates the unmet need to develop more effective therapeutic strategies that, not only control the disease biochemically, but also improve patient well-being. In addition to ACROQOL, the only validated acromegaly questionnaire, we specifically assessed fatigue, an acromegaly related typical symptom, using FSS, a questionnaire that had never been studied previously in this patient group. The survey gives us insight on how a symptom could impact QOL, which was worse than in normal controls, and highlights the importance of assessing symptoms when studying treatment interventions in acromegaly. The third questionnaire we used was SF36, which is the most widely used QOL assessment tool globally and has been used frequently in acromegaly [11, 12].

QOL in patients with acromegaly is a concern. As first-line treatment to achieve remission in acromegaly, surgery is recommended as it can achieve higher remission rates compared with first line medical treatment (66% vs 45%) [10]. For those unable to achieve remission by surgery, long term medical treatment is the mainstay therapeutic strategy besides radiotherapy and a significant number of patients do achieve biochemical remission on maximal treatment, sometimes needing combination regimes [9]. Conversely long-term biochemical cure is not usually associated with normal life quality as highlighted by our findings. This has been shown by a number of studies but in view of the rarity of the disease most have used heterogenous groups and been cross-sectional as opposed to prospective [6]. Study populations vary within studies differing in treatment stage, disease control, length of remission and treatment received. This makes data less accurate and at times difficult to interpret. Unlike these studies we tried to create study groups which, apart from treatments received, were less heterogenous and studied sometime after surgery. Our groups did not differ in age, duration of disease from diagnosis, presence of hypopituitarism, presence of metabolic complications and mental health issues. Irrespective of this all our groups still achieved a QOL which is lower than the normal population.

Data from older longitudinal studies comparing QOL in surgical and medical treatment only (i.e., no surgery) have shown that QOL in both groups improves from baselines but is not different between the groups at 12 and 48 weeks [19, 20]. In our study, however, we excluded the few patients who required only medical treatment for acromegaly. Most of these patients had either inoperable tumours or were not medically fit enough to undergo surgery. These factors, therefore, would have confounded the QOL scores and besides in clinical practice, the vast majority of patients would first undergo surgery in line with the guidelines.

A Japanese study from 2014 also compared the QOL between surgical and medical patients [18]. This study was a cross-sectional study and showed that the QOL in surgical patients (n = 12) was better than those who were medically treated (n = 14). The authors also postulated that radiotherapy played an important role in lowering QOL scores in medical patients, as the difference between QOL scores was less significant once those with radiotherapy were excluded (n = 4) from the medical group. The results from our study are dissimilar to these findings however our study had more than twice the number of subjects. Besides, it is not clear from their published results, how many of the patients in the medical group also had surgery. This along with the differences in ethnicity between these two studies could possibly explain the dissimilarity in the results. In addition, we were also unable to confirm their finding with regards to radiotherapy, as removing radiotherapy patients from both groups in our study didn’t alter the results, and the QOL scores remained comparable (supplementary table 1).

We have also found that medically controlled patients have better physical scores: SF36 PCS and possibly, ACROQOL physical subdomain. This could be related to the improvement in external features of the disease, better control of physical symptoms, or perhaps due to a sense of physical well-being from frequent monitoring and reassurance. Higher proportion of men in the medically controlled group, could have been a contributing factor since QOL scores in the general population tend to be worse in females [27] and in acromegaly QOL is affected more in females due to delay in diagnosis and added comorbidities [28] and more socioeconomic burden of the disease [29]. However, since the difference was still significant adjusting for gender, it was not a confounding factor in our study.

The reason for a low QOL in acromegaly patients despite treatment is debatable, but there are several contributing factors. Despite substantial improvements in healthcare in the recent past, there is still considerable delay in diagnosis of acromegaly, with some studies estimating the delay to be around 8–10 years from the onset of symptoms [30]. Owing to this delay severe irreversible damage such as changes in joint, soft tissues, voice, and physical appearance have already occurred by the time of diagnosis. Hence treatment, at best, can only slow down the progression of these changes, as the damage has already taken place [31]. There is an increased incidence of other chronic conditions in acromegaly which impair QOL such as depression [32], high BMI [33], type 2 diabetes [34]. Despite treatment, these conditions continue to progress in most cases, further deteriorating QOL. Some treatment options or resulting consequences could also be a contributing factor such as radiotherapy [35, 36] or hypopituitarism [36] both of which have been noted to reduce QOL in some studies. However, the systematic review by Geraedts et al. [6] didn’t confirm the link between hypopituitarism and QOL. An interesting but unproven hypothesis is that current criteria for disease remission rely heavily on post treatment IGF-1 or GH levels. This may not reflect the true tissue exposure of GH and therefore may have a role to play in persistent low QOL in acromegaly despite treatment.

One of the main limitations of our study is the cross-sectional design and therefore absence of longitudinal data and control group. However, the latter was countered by including QOL data from large population cohorts. Secondly, our sample size was relatively small which may have decreased the statistical power. Also, the BMI data for the patients in this study was not available to us, which could be a potential confounding factor. Long-term, large prospective interventional studies looking at effects of different therapeutic strategies on symptoms and QOL are necessary to confirm our findings.

In conclusion, while surgery is superior in achieving biochemical remission in acromegaly, we have shown that medical treatment achieves similar QOL. In patients who do not achieve biochemical remission after surgery, medical treatment not only maintains QOL comparable to surgical patients in remission, but may also be associated with better QOL in physical subdomain. Secondly, compared to healthy controls, the QOL remains worse in acromegaly irrespective of the type of treatment. Strategies to diagnose acromegaly earlier and novel treatment modalities are required to improve this important patient-related health outcome.

## Supplementary Information

Below is the link to the electronic supplementary material.

Supplementary file1 (DOCX 17 kb)

## Data Availability

Available on request.
