# Supplementary material for: Surgically treated acromegaly patients have a similar quality of life whether controlled by surgery or requiring additional medical therapy (QuaLAT Study)
Source: Pituitary. 2021 May 12;24(5):768–77. doi: 10.1007/s11102-021-01153-4 (PMC8416856; doi:10.1007/s11102-021-01153-4)
Supplement: Supplementary file 1 — Supplementary file1 (DOCX 17 kb) [file 11102_2021_1153_MOESM1_ESM.docx]

**Supplementary table 1: Comparison of QOL scores between surgical and medical group after removing radiotherapy patients (n=9), calculated using ANOVA and Fisher’s least significance difference (LSD) test for post-hoc analysis**

|  | Group 1 (Surgical)  n=29 | Group 2 (Medical)  n=19 | Mean difference (95% CI) | P value |
| --- | --- | --- | --- | --- |
| **Mean total ACROQOL ± SD** | 50.4 ± 28.1 | 54.5 ± 26.4 | -4.1  (-20.3 to 12.2) | 0.62 |
| **ACROQOL physical domain** | 46.8 ± 29.3 | 56.1 ± 30.4 | -9.3  (-27.4 to 8.8) | 0.31 |
| **ACROQOL psychological/ appearance domain** | 48.3 ± 31.0 | 43.2 ± 25.4 | 5.1  (-11.9 to 22.1) | 0.55 |
| **ACROQOL psychological/ personal relations %** | 62.1 ± 29.0 | 63.9 ± 28.7 | -1.8  (-18.9 to 15.2) | 0.83 |
| **FSS mean score ± SD** | 4.3 ±2.2 | 4.3± 2.2 | -0.1  (-1.4 to 1.2) | 0.93 |
| **SF36 Physical component score (PCS)** | 41.0 ± 10.7 | 45.6 ± 12.3 | -4.6  (-11.6 to 2.3) | 0.19 |
| **Physical functioning (PF)** | 56.9 ± 30.7 | 69.2 ± 31.1 | -12.3  (-30.9 to 6.3) | 0.19 |
| **Role-Physical (RP)** | 48.4 ± 30.5 | 65.1 ± 34.1 | -16.7  (-36.3 to 2.9) | 0.09 |
| **Bodily pain (BP)** | 50.1 ± 23.3 | 60.8 ± 30.1 | -10.8  (-26.5 to 4.9) | 0.18 |
| **General health (GH)** | 41.3 ± 27.0 | 48.1 ± 33.2 | -6.7  (-25.2 to 11.8) | 0.47 |
| **SF36 Mental component score (MCS)** | 39.0 ± 12.4 | 43.0 ± 16.3 | -4.0  (-12.7 to 4.6) | 0.36 |
| **Role-Emotional (RE)** | 55.2 ± 31.7 | 67.1 ± 35.2 | -11.9  (-31.7 to 7.9) | 0.23 |
| **Vitality (VT)** | 36.2 ± 22.9 | 42.1 ± 30.4 | -5.9  (-22.5 to 10.6) | 0.48 |
| **Mental health (MH)** | 52.3 ± 25.4 | 62.2 ± 30.3 | -9.8  (-26.7 to 7.2) | 0.25 |
| **Social functioning (SF)** | 57.8 ± 31.1 | 69.8 ± 33.2 | -12.0  (-30.9 to 7.0) | 0.21 |
